# Probing Neurovisceral Integration via Functional Near-Infrared Spectroscopy and Heart Rate Variability

**DOI:** 10.3389/fnins.2020.575589

**Published:** 2020-11-25

**Authors:** Emma E. Condy, Bruce H. Friedman, Amir Gandjbakhche

**Affiliations:** ^1^National Institute of Child Health and Human Development, Bethesda, MD, United States; ^2^Department of Psychology, Virginia Tech, Blacksburg, VA, United States

**Keywords:** respiratory sinus arrhythmia, behavioral inhibition, neurovisceral integration, functional near-infrared spectroscopy, central autonomic network

## Abstract

The neurovisceral integration model (NVM) proposes that an organism’s ability to flexibly adapt to its environment is related to biological flexibility within the central autonomic network (CAN). One important aspect of this flexibility is behavioral inhibition (Thayer and Friedman, 2002). During a behavioral inhibition task, the CAN, which comprises a series of feedback loops, must be able to integrate information and react to these inputs flexibly to facilitate optimal performance. The functioning of the CAN is shown to be associated with respiratory sinus arrhythmia (RSA), as the vagus nerve is part of this feedback system. Although the NVM has been examined through neural imaging and RSA, only a few studies have examined these measures simultaneously during the neuroimaging procedure. Furthermore, these studies were done at rest or used tasks that were not targeted at processes associated with the NVM, such as behavioral inhibition and cognitive flexibility. For this reason, the present study assessed RSA and neural activation in the pre-frontal cortex simultaneously while participants completed a behavior inhibition task. RSA and functional near-infrared spectroscopy were collected in 38 adults, and resting levels of pre-frontal activation were negatively related to RSA, but pre-frontal activation during the behavior inhibition task was not. The negative relationship between RSA and oxygenated hemoglobin is consistent with previous functional magnetic resonance imaging work examining the NVM at baseline and should be further studied. Additional research investigating how this relationship may change based on task demands or environmental contexts would help clarify the applicability of the model.

## Introduction

The principal role of the autonomic nervous system (ANS) is to maintain the body’s internal environment by distributing and integrating specific signals to target organs (Janig and Habler, 2000). The ANS is traditionally divided into two branches: the parasympathetic and sympathetic, which have anatomical, functional, and neurochemical distinctions^1^. The neurovisceral integration model (NVM) proposes that these systems are part of the central autonomic network (CAN) (Thayer and Lane, 2000), which operates through a series of feedback loops within the central nervous system. These circuits include both central and peripheral inputs and outputs and subsequently influence several behavioral and physiological processes. Importantly, measurements of the peripheral outputs of this system can be used to index the functionality of feedback loops in the CAN. Cortical structures in the CAN that are highlighted in the NVM include the medial pre-frontal cortex (mPFC), anterior cingulate cortex, and the insula (Benarroch, 1993).

The NVM proposes that cortical structures such as the mPFC institute tonic inhibition on activation of the amygdala, resulting in heightened CAN output (Thayer and Lane, 2009). This view is based on evidence from animal and human neuroimaging studies, which have established structural interconnections, and pharmacological studies in which both branches of the ANS are blocked and show increased activation compared with the normal state, indicating that the system is under the tonic inhibitory influence (Thayer and Lane, 2009; Thayer et al., 2012). One of the outputs of the CAN is the 10^*th*^ cranial, or vagus nerve, which provides parasympathetic input to the heart. This vagal activation can be indexed through heart rate variability (HRV), which is commonly used to provide information about the functionality of the CAN.

The variation from beat-to-beat in heart rate, known as HRV, is controlled by sympathetic and parasympathetic nervous system inputs to the sinoatrial node of the heart (Task Force, 1996). Differences in the temporal dynamics of the primary neurotransmitters involved in sympathetic and parasympathetic activation (norepinephrine and acetylcholine, respectively) allow fast changes in interbeat interval (IBI) length to be attributed to vagal control (Saul, 1990). Respiratory sinus arrhythmia (RSA) refers specifically to changes in the lengths of IBIs as a result of respiration (Berntson et al., 1993), which can be quantified through changes within the high-frequency band (0.12–0.40 Hz), known as high-frequency heart rate variability (HF-HRV) (Allen et al., 2007), or through a time-based metric using the root mean square successive difference (RMSSD) (Berntson et al., 1997; Laborde et al., 2017).

In the NVM, high baseline RSA and increased RSA reactivity are viewed as indicative of increased flexibility within the CAN (Friedman, 2007). Flexibility within this biological system is manifested through greater cognitive and behavioral flexibility, as demonstrated by skills such as better emotion regulation and inhibitory capacities (Thayer et al., 2009). The present study aimed to further investigate the tenets of the NVM through simultaneous measurement of RSA and cortical activation. Although previous studies have looked at RSA in relation to brain activity, there are many limitations in this literature, such as small sample sizes and flawed methodology. In the present study, pre-frontal cortex activity was assessed via functional near-infrared spectroscopy (fNIRS) in concert with RSA during a baseline condition and a cognitive task. Although functional imaging has been used to examine this model during resting state in previous studies, the present study investigated whether the tenets of the NVM are upheld across multiple contexts using a more ecologically valid neural metric to do so. As such, the present study can be viewed as a “proof of concept” investigation; i.e., it is the implementation of a method to demonstrate its feasibility (Schmidt, 2006).

### Human Neuroimaging and the Neurovisceral Integration Model

Much of the literature supporting the NVM and the relationship between RSA and the CAN is based on animal models and blockade studies. However, there have been several human neuroimaging studies examining the theory. These studies were reviewed in two functional magnetic resonance imaging (fMRI) meta-analyses, which concluded that a number of areas associated with the NVM were activated in conjunction with heightened HF-HRV (Thayer et al., 2012; Beissner et al., 2013). Specifically, HF-HRV was associated with the activation of regions of the mPFC (Thayer et al., 2012).

Furthermore, Beissner et al. (2013) note that many of the structures associated with HF-HRV in their meta-analysis are part of the *default mode network*, a network of brain regions that are active when an individual is not engaged in a task and is instead focused on internal events (see Raichle, 2015 for a review). Some of these regions overlap with the CAN, such as the ventromedial pre-frontal cortex and dorsomedial pre-frontal cortex, posterior cingulate cortex, and hippocampal formation (Buckner et al., 2008). Indeed, a recent review paper on the NVM has implicated networks such as the executive control and default mode networks in the hierarchical structure of neurovisceral integration (Smith et al., 2017). However, the majority of studies included in these meta-analyses had limited sample sizes (Beissner et al., 2013, sample size median = 12, range = 4–41; Thayer et al., 2012, sample size median = 15, range = 6–93), used dissimilar methods (e.g., some baseline and others task-based measures), and incorporated HF-HRV measurements that were taken at a different time than the neuroimaging data. These are all significant limitations, but the last point is particularly concerning. It was assumed in these studies that the participants’ state during an independent baseline HF-HRV measurement would be comparable with their state during the neuroimaging data collection. Simultaneous collection of HF-HRV and neuroimaging data is necessary to avoid such assumptions, which cannot be unequivocally made. Regardless, both meta-analyses indicated that baseline HF-HRV values correlate with neural activity in areas implicated in the CAN.

These limitations were noted and addressed in subsequent research aimed at improving the examination of the NVM. In two studies that had significantly larger samples, associations were found in similar brain regions (left insula, right hippocampus, and right superior temporal gyrus) to these meta-analyses (Allen et al., 2015; Jennings et al., 2015). However, HF-HRV data were still collected outside of the scanner, approximately 2 weeks before neuroimaging. Furthermore, these studies used resting-state data, whereas many of the studies in the meta-analyses used task reactivity. These authors argued that the relationship between RSA and neural activation would be inverse during the resting state because many previous studies examined RSA reactivity in relation to neural activation. The logic behind this argument is that because higher resting HF-HRV is associated with larger reductions in task HF-HRV (a negative correlation) and larger reductions in task HF-HRV are associated with less neural activation (a positive correlation), then the transitive property suggests that higher resting HF-HRV is associated with less neural activation (Allen et al., 2015). Their results supported this hypothesis; activation of multiple structures implicated in the NVM (e.g., insula, amygdala, pre-frontal cortex) was negatively related to HF-HRV.

Other studies have improved the co-examination of RSA and fMRI by using time series analyses that look at the two signals simultaneously. Through comparison of sliding-window calculations of both resting fMRI and HRV, greater functional *connectivity* between the dorsal anterior cingulate cortex/amygdala and the mPFC, as well as the insula, was associated with heightened HF-HRV (Chang et al., 2013). Although these results are compelling, the use of connectivity analyses (vs. regional activation) as the dependent variable may have missed other regions implicated by the NVM. Using a similar method, positive correlations between HF-HRV and several areas associated with the NVM including the amygdala, right dorsal mPFC, and right dorsal lateral PFC, as well as negative correlations with the left posterior insula and right medial temporal gyrus during a motor task, have been found (Gianaros et al., 2004). In the same study, during a series of memory tasks, HF-HRV was positively correlated with the left insula and amygdala–hippocampal complex and right ventromedial PFC and cerebellum. These findings support the NVM by examining CAN activation and RSA simultaneously, but they do not probe the model in terms of simple regional activation at rest; they either used functional connectivity metrics or did not include resting state. Extending this method to include resting-state measurement and a task that taps into the psychological constructs implicated in the NVM, such as behavioral inhibition, will better elucidate how the CAN functions, particularly when being actively recruited.

One way to test behavioral inhibition is the go/no-go (GNG) task, which was developed for this purpose (Donders, 1969). The GNG has been used across a variety of studies to assess how behavioral inhibition relates to other functions. GNG performance is attenuated in many disorders characterized by inhibition deficits, such as attention deficit–hyperactivity disorder (Dillo et al., 2010), obsessive–compulsive disorder (Lee et al., 2009), and autism spectrum disorder (Uzefovsky et al., 2016). The task consists of two types of stimuli: the “go” stimulus, which indicates that the subject should complete the behavioral response (e.g., a button press), and the “no-go” stimulus, which indicates that the subject should not complete the behavioral response (e.g., no response). By prompting a prepotent response through a series of “go” trials, the subject is then challenged to inhibit the response behavior during the “no-go” stimulus. Its wide use in the neuroimaging literature provides extensive information about the neural correlates of behavioral inhibition, which is implicated in the NVM. Meta-analyses on fMRI studies of the GNG show increased activity in frontal cortical areas during “no-go” trials, such as the bilateral mPFC (Watanabe et al., 2002), the pre-supplementary motor area (Mostofsky et al., 2003), and the right inferior parietal lobule (Swick et al., 2011). The mPFC is one of the structures in the CAN, making the GNG task an appropriate paradigm for the present study. However, due to temporal and ecological limitations of fMRI technology, we incorporated a different imaging modality (i.e., fNIRS) to assess inhibition in the GNG task.

### Functional Near-Infrared Spectroscopy

fNIRS is an optical imaging technique that projects infrared light (650–1,000 nm) from a source diode into tissue and measures the backscatter of this light using detectors. Based on the amount of backscatter, the concentration of oxygenated hemoglobin (O_2_Hb) and deoxygenated hemoglobin (HHb) can be determined because of their different optical properties (Fox and Raichle, 1986; Ferrari and Quaresima, 2012). Not only does fNIRS have better temporal resolution than fMRI, but there is also less concern about motion artifact interfering with the signal, affording greater ecological validity and task flexibility (Irani et al., 2007; Lloyd-Fox et al., 2010). These are important considerations in the context of the NVM, in which anxiety and stress can be triggered by neuroimaging approaches such as fMRI (Eatough et al., 2009; Lueken et al., 2012). These states are associated with the activation of CAN structures, which confounds the external validity of conclusions from such studies. Additionally, the NVM has yet to be evaluated in populations with compliance issues in other neuroimaging environments, such as children and those with neurodevelopmental disorders (Yerys et al., 2009), making the use of fNIRS advantageous. For these reasons, fNIRS is a valuable technique for collecting neuroimaging data from the cortex during task-based studies of cognition and behavior that can be applied to the NVM.

Similar to fMRI findings, fNIRS studies have shown increased pre-frontal O_2_Hb during the GNG task compared with rest (Anderson et al., 2014) and increased O_2_Hb during “no-go” trials compared with “go” trials in lateral pre-frontal locations (Herrmann et al., 2005). However, these studies simply looked at neural activation between trial types. When actual responses to the GNG task are considered, successful behavioral inhibition during “no-go” trials predicts lower O_2_Hb in the mPFC but not in lateral locations (Rodrigo et al., 2014). Together, these findings indicate that increased activation in frontal lateral areas (such as the inferior frontal gyrus) may be associated with the presentation of the “no-go” condition or behavioral inhibition opportunity itself, but that successful behavioral inhibition appears to specifically be associated with decreased activity in the mPFC. This distinction may account for discrepancies across the fMRI GNG studies. Furthermore, the NVM posits that the mPFC is integral in the top–down, tonic inhibition of the CAN. Previous work suggests this may be reflected by a negative relationship between activation of the PFC and RSA at rest (Allen et al., 2015). However, when the mPFC is recruited during an active behavior inhibition task, the relationship between its activation and RSA may differ. The present study aimed to incorporate simultaneous fNIRS and RSA measurements throughout a baseline period, as well as during the GNG task, to assess their relationship as described in the NVM across various contexts. We hypothesized that RSA and neural activation in areas contained in the CAN, namely the mPFC, will be related at rest and during the GNG task.

## Materials and Methods

### Participants

Participants were recruited through the healthy volunteer database at the National Institutes of Health in Bethesda, MD, and were compensated for participation in the study. Exclusionary criteria included: past or present vascular disease, skin disease, or any history of head injury, cardiovascular disease, or congenital heart condition, seizure, or stroke. A total of 45 participants were brought to the lab to participate in the study. Participants completed a health history questionnaire that screened for psychiatric diagnoses; one indicated a current psychiatric diagnosis and was removed from the sample. Furthermore, due to data loss in either the fNIRS or electrocardiogram (ECG) signals, 38 participants were retained for analyses in the present paper. The final sample consisted of 38 healthy adults (age *M* = 37.18, *SD* = 14.67). The majority of participants were right-handed (32; 84.2%), three were left-handed, and three were ambidextrous as measured on the Edinburgh Handedness Inventory (Oldfield, 1971). These participants were retained in the analyses because we did not expect a lateralization effect for pre-frontal activation at rest or during the task. The present study was part of an ongoing research protocol that was approved by the National Institute of Child Health and Human Development’s Institutional Review Board (NCT01212029). All participants underwent the informed consent procedure as approved by the Institutional Review Board before participating in the study.

### Measures

#### Electrocardiogram

ECG was collected through the BioPac MP160 system, outfitted with an ECG amplifier (ECG100C). The ECG was sampled at 1,000 Hz with an amplifier gain of 1,000, a low pass filter set at 35 Hz, and high pass filters set at 0.5 Hz. The signal was recorded through AcqKnowledge 4.4 software (BioPac Systems Inc.). A Lead II ECG configuration was used, which requires Ag-AgCl spot electrodes to be placed underneath the left collar bone on the chest and underneath the rib cage on the right side. RSA was then derived from this signal through Kubios HRV software (Tarvainen et al., 2014) using the time-domain RMSSD metric. RMSSD is shown to reflect cardiac vagal tone (Laborde et al., 2017) and can be derived from short recording periods such as the 30-s windows used in the present study (Munoz et al., 2015).

#### Functional Near-Infrared Spectroscopy

Neural activity was measured through a continuous wave fNIRS device (fNIR Devices LLC), which emits light at two wavelengths (730 and 850 nm) and samples at a rate of 2 Hz. The use of two infrared light wavelengths allows both oxy- and deoxy-hemoglobin levels to be measured, in which they produce differential amounts of backscatter to be picked up by the detectors. The system uses 4 sources and 10 detectors spaced 2.5 cm from another, creating a 16-channel silicone headband placed across the forehead of participants to measure pre-frontal activity. The headband was centered at Fpz. Data from the device were collected through COBI Studio software (Ayaz et al., 2011).

### Procedure

The protocol for this study was approved by the *Eunice Kennedy Shriver* National Institute of Child Health and Human Development’s Institutional Review Board. Participants were instructed to abstain from alcohol for 24 h, caffeine for 6 h, and vigorous exercise for 2 h before their data collection session. All participants were scheduled for their session to begin between the hours of 8:15–11:30 am. Upon entering the lab, the subject was provided a copy of the informed consent and reviewed it with the researcher. Once subjects provided consent, they were outfitted with ECG electrodes and a respiration monitor. The ECG and respiration signal were then examined to ensure that the physiological equipment was properly and securely applied. Once this was completed, the subject filled out the series of behavioral scales. These questionnaires took approximately 15 min to complete, after which the fNIRS headband was applied, and signals were checked to ensure that the headband had been properly applied. The physiological and neural imaging portion of the session then began. Participants were positioned in front of a computer equipped with E-Prime 2 Stimulus Presentation software (Schneider et al., 2002), which presented all instructions and tasks for the remainder of the data collection session.

#### Baseline

After completing the health history questionnaire and behavioral scales and application of the fNIRS headband, a 6:30 min recording period began. During this time, participants were instructed to sit quietly while watching a mildly stimulating video (Coral Sea Dreaming: Plankton Productions & MJL Network, 2014) presented on a computer monitor, consistent with “vanilla” baseline guidelines (Jennings et al., 1992). Such baselines are common in psychophysiology because they maintain minimal engagement and create more uniform conditions across participants than a traditional baseline. During this time, physiological and neural imaging data were collected.

#### Cognitive Task

Participants then completed two versions of a GNG task: the simple GNG and the emotional GNG, the order of which was counterbalanced across participants. Only data from the Simple GNG task are analyzed for the purposes of this study. The emotional GNG was not included due to issues with performance on the task for a number of participants (e.g., participants had difficulty understanding or remembering the emotional GNG directions). The simple GNG paradigm uses basic stimuli (e.g., numbers, letters, shapes) to examine behavioral inhibition abilities. In the present study, a GNG paradigm using letter stimuli was presented to participants through E-Prime 2. The timing and proportion standards for both the simple GNG and emotional GNG in this study were modeled after (Schulz et al., 2007), in which both the simple and emotional GNG task were used. Each block consisted of 192 total trials, which were comprised of 75% “go” stimuli (the letter “Y”) and 25% “no-go” stimuli (the letter “X”), resulting in 144 “go” and 48 “no-go” stimuli. Each stimulus was presented for 500 ms, followed by a pseudorandom interstimulus interval of 1,500 ± 250 ms. The interstimulus interval jitter is used to reduce the effect of stimulus anticipation of the subject’s reaction time. With these timing parameters, the task was approximately 6 min and 24 s long. The use of a block design best served the study’s aim, in which it allowed for a sliding window analysis to be conducted throughout the task procedure to examine the relationship between the fNIRS and RSA metrics. Although this design does not provide a pure period of active behavioral inhibition, it draws upon behavior inhibition resources during the task period compared with baseline. On-screen instructions informed the participants that they would be presented with a series of letters. They were instructed to press the space bar when they saw the letter “Y” and not to press any buttons when they saw the letter “X.” If they understood these instructions, they were instructed to press the space bar, which initiated six task practice trials. The task began upon completion of these trials.

### Data Processing

#### Pre-processing Electrocardiogram

All ECG data were preprocessed using AcqKnowledge 4.4 software. First, event markers sent from the EPrime script were identified in the ECG file, and their times were recorded for use in calculating the sliding window parameters. Next, the “Find Cycle” function was used to automatically detect and mark R-spikes in the ECG signal. These marks were then visually inspected to ensure that aberrations in the signal were not mistakenly marked as R-spikes and that R-spikes were not missed by the algorithm. The “Find Cycle” function was used again to locate these visually inspected marks and then calculate the time between each R-spike pair (i.e., the IBI) in milliseconds. These values were then saved as a text file that contained two columns: time and IBI length (millisecond).

#### Pre-processing Functional Near-Infrared Spectroscopy

Imaging data were preprocessed by the subject through HOMER2 software to remove motion artifact and physiological noise. The parameters used within each function are defined in Table 1. First, event markers sent from the EPrime script in each file were located and their times recorded for use in the sliding window parameter calculations. The raw light intensity files were converted to the HOMER2 file format (.nirs). The raw light intensity was converted to optical density (function: *hmrIntensity2OD*), and bad channels were removed (function: *enPruneChannels*, dRange = 500–4,000, SNRthresh = 2, SDrange = 0–45). A wavelet transform was used to correct for motion artifact (function: *hmrMotionCorrectWavelet*) using the default interquartile range (0.1), as this is optimal for motion correction (Brigadoi et al., 2014). Any remaining motion artifact was then removed through the motion artifact detection tool (function: *hmrMotionArtifact*, tMotion = 0.5, tMask = 2.0, STDEVthresh = 20, AMPthresh = 0.5). The signal was then bandpass filtered (function: *hmrBandpassFilt*, hpf = 0.010, lpf = 0.50) to remove baseline drift and physiological noise. Finally, the optical density signal was converted to hemoglobin concentration (function: *hmrOD2Conc*) by applying the Modified Beer–Lambert Law. Depending on the wavelengths of light that are used, the Modified Beer–Lambert Law allows for the calculation of oxygenated and deoxygenated hemoglobin concentration (micrometer) in a highly scattering medium, such as biological tissues, by accounting for scattering losses and a longer optical path length because of scattering. The O_2_Hb values were then saved as text files for each subject. Finally, the O_2_Hb time series for each subject was z-scored by channel.

**TABLE 1:** Homer2 fNIRS pre-processing parameter definitions.

Function name	Parameter name	Definition
*enPruneChannels*	dRange	Allowable optical density (OD) range
	SNRthresh	Minimum signal-to-noise ratio allowable
	SDrange	Maximum standard deviation allowable
*hmrMotionArtifact*	tMotion	Time range to check for a motion artifact (seconds)
	tMask	Amount of time (seconds) surrounding a detected motion artifact where data are to be removed
	STDEVthresh	Threshold for a change in signal standard deviation within the tMotion period to be marked as motion artifact
	AMPthresh	Threshold for a change in signal amplitude within the tMotion period to be marked as motion artifact
*hmrBandpassFilt*	hpf	High pass filter cutoff (Hz)
	lpf	Low pass filter cutoff (Hz)

#### Post-processing Electrocardiogram and Functional Near-Infrared Spectroscopy

Event markers from each subject’s ECG and fNIRS data files were used to calculate the parameters for sliding window epochs to be used for subsequent analyses. The window length was set to 30 s with 7 s of overlap with adjacent windows, resulting in a 46.67% overlap between adjacent windows. For each condition, this resulted in 16 windows, for a total of a 6:15 min condition period. This was done to generate start and stop times for each window within each condition for both the ECG and fNIRS signals. The window values from the ECG signal were then used to compute RMSSD for each 30 s window (Takahashi et al., 2017) in Kubios HRV software from the IBI files previously mentioned. The corresponding window values from the fNIRS signal markers were then used to compute the mean of the standardized O_2_Hb signals within each window for each channel. The corresponding RMSSD and O_2_Hb values were then used to complete subsequent analyses for each subject.

### Analyses

RMSSD and O_2_Hb concentrations were quantified over the baseline period through sliding window analysis. Doing this allowed a series of RMSSD and O_2_Hb values to be generated at multiple time points over the baseline period so that the correspondence between the RMSSD and neural values could be tracked over the baseline time course for each subject. These analyses were modeled after those conducted by Chang et al. (2013), who performed similar analyses to compare RMSSD and fMRI signals. These values were standardized and entered in a subject-level general linear model to determine whether RMSSD predicted O_2_Hb concentration. This was done for each subject at each channel, providing 16-channel parameter estimates per subject per condition to be used for statistical analyses in subsequent steps. Running these models for each channel for each subject yielded a 38 (subject) by 16 (fNIRS 0channels) matrix of standardized parameter estimates (β) for each condition. A series of one-sample *t*-tests were then conducted to determine whether the relationship between RMSSD and O_2_Hb was different than 0 across the 16 channels. If a test was significant, this would indicate that the relationship between RMSSD and activation at that channel was not equal to 0 and would support the hypothesis that activation at that specific region of the pre-frontal cortex was related to RMSSD. These processes and statistical tests were conducted through R software (R Core Team, 2017).

## Results

### Baseline

**FIGURE 1:**
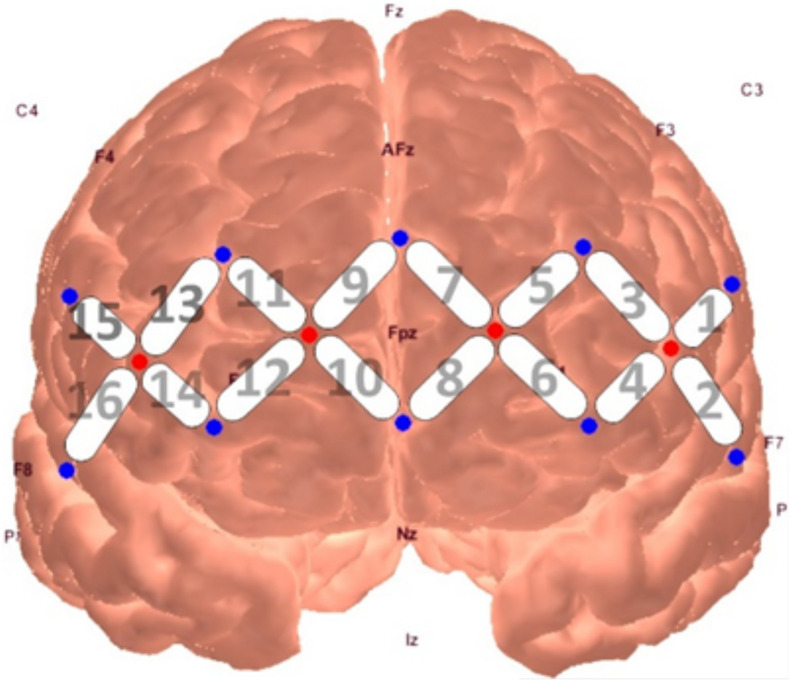
fNIRS probe placement and channel locations rendered in AtlasViewer software (Aasted et al., 2015).

A series of non-parametric one-sample *t*-tests were conducted using standardized β weights from the subject-level general linear models where RMSSD predicted O_2_Hb during the baseline condition as the dependent variable. At *p* < 0.05, increased RMSSD predicted lower O_2_Hb levels at channel 1 (*V* = 60, *p* = 0.030*)*, channel 10 (*V* = 179, *p* = 0.008*)*, and channel 15 (*V* = 107, *p* = 0.049*)* during baseline. The channel configuration across the PFC can be seen in Figure 1. The resulting statistics from the series of tests for the baseline condition are summarized in Table 2. None of these tests were significant after using the Holm–Bonferroni method of sequentially rejective *t*-tests.

**TABLE 2:** One sample *t*-tests of standardized regression coefficients of RMSSD predicting O_2_Hb during baseline and the GNG task.

	Baseline							Go/No-Go						
Channel	*M*	*SD*	*V*	*n*	*p*	*d*	95% CI_*d*_	*M*	*SD*	*V*	*n*	*p*	*d*	95% CI_*d*_
1	–0.14	0.24	60	22	0.030*	–0.58	[−1.03, −0.13]	0.01	0.29	104	20	0.985	0.03	[−0.41, 0.47]
2	–0.05	0.22	204	33	0.177	–0.23	[−0.58, 0.12]	0.02	0.23	231	30	0.984	0.09	[−0.27, 0.45]
3	–0.04	0.22	219	32	0.410	–0.18	[−0.53, 0.17]	−0.01	0.28	235	30	0.968	−0.04	[−0.40, 0.32]
4	–0.06	0.21	184	32	0.139	–0.29	[−0.64, 0.06]	0.03	0.26	254	30	0.670	0.12	[−0.24, 0.48]
5	–0.01	0.20	217	28	0.762	–0.05	[−0.42, 0.32]	−0.02	0.21	120	25	0.263	−0.1	[−0.49, 0.29]
6	–0.04	0.25	302	37	0.464	–0.16	[−0.48, 0.16]	0.00	0.24	296	34	0.987	0	[−0.34, 0.34]
7	–0.03	0.22	319	37	0.633	–0.14	[−0.46, 0.18]	−0.03	0.23	257	34	0.499	−0.13	[−0.47, 0.21]
8	–0.06	0.23	238	36	0.139	–0.26	[−0.59, 0.07]	−0.02	0.24	263	33	0.764	−0.08	[−0.42, 0.26]
9	–0.07	0.26	219	36	0.074	–0.27	[−0.60, 0.06]	0.00	0.25	303	34	0.933	0	[−0.34, 0.34]
10	–0.12	0.27	179	37	0.008*	–0.44	[−0.78, −0.10]	0.01	0.21	322	34	0.685	0.05	[−0.29, 0.39]
11	–0.05	0.23	223	32	0.454	–0.22	[−0.57, 0.13]	−0.03	0.20	163	30	0.158	−0.15	[−0.51, 0.21]
12	–0.06	0.21	221	35	0.127	–0.29	[−0.63, 0.05]	0.00	0.21	277	33	0.958	0	[−0.34, 0.34]
13	–0.06	0.22	237	35	0.207	–0.27	[−0.61, 0.07]	0.02	0.26	293	33	0.832	0.08	[−0.26, 0.42]
14	–0.03	0.25	306	36	0.681	–0.12	[−0.45, 0.21]	0.01	0.25	304	34	0.919	0.04	[−0.30, 0.38]
15	–0.12	0.24	107	27	0.049*	–0.50	[−0.90, −0.10]	0.01	0.20	163	24	0.726	0.05	[−0.35, 0.45]
16	–0.09	0.23	192	34	0.072	–0.39	[−0.74, −0.04]	0.06	0.25	345	32	0.134	0.24	[−0.11, 0.59]

The summarized statistical tests include *uncorrected p*-values Where, Cohen’s $d=\frac{M_{i}}{S\,D_{i}},C\,I_{d}=d\pm \left(z_{c\,r\,i\,t}*S\,E_{d}\right),a\,n\,d\,S\,E_{d}=\sqrt{\frac{1}{n_{i}}+\frac{d^{2}}{2*n_{i}}}$ (Turner and Bernard, 2006). *p < 0.05.

### Go/No-Go Task

First, to verify that the GNG task had the intended effect of increasing pre-frontal activation, the average O_2_Hb concentrations over the baseline and GNG task period were compared. Although channel-level activation may show an interaction effect across tasks, such analyses were outside the purview of the present study because the hypothesis was only directed at the relationship between pre-frontal activation and RSA. Thus, the mean O_2_Hb level during baseline across all channels was averaged by the subject. The same was done for O_2_Hb levels during the GNG task. This provided each subject with one O_2_Hb measure that indicated global pre-frontal activation at baseline and during the GNG task. Three subjects were missing data during the GNG task and thus were not included in this analysis (*n* = 35). These values were compared using a paired samples *t*-test and indicated that the mean (*M*) O_2_Hb across the pre-frontal cortex was different between the baseline and GNG task [*t*(34) = −6.45, *p* < 0.001], such that O_2_Hb was higher during the GNG task (*M* = 0.011, *SD* = 0.023) than baseline (*M* = −0.024, *SD* = 0.025). These results indicate that the GNG task did elicit greater pre-frontal activation than during a resting baseline.

To examine whether the relationship between pre-frontal activation and RMSSD seen during baseline held during a behavioral inhibition task, the same set of analyses as described previously were conducted for the standardized β weights derived from the GNG task. None of the tests were significant at the *p* < 0.05 level, indicating that RMSSD did not predict pre-frontal activation during the GNG task. The results of these tests are summarized in Table 2.

## Discussion

Consistent with the NVM, the results of this study support the hypothesis that RSA is related to pre-frontal activation at rest. Although these findings were not maintained when corrected for multiple comparisons, the relationship between baseline RSA and pre-frontal activation in this study was consistent with previous NVM studies. For example, standardized beta weights were significantly different than 0 (at *p* = 0.05) at various sites across the PFC during baseline, which reflects a predictive relationship between RSA and PFC activation. Notably, this was observed at channel 10, which is located over the right mPFC, one of the CAN structures implicated in the NVM (Gianaros et al., 2004; Thayer et al., 2012). The non-significant relationship between RSA and O_2_Hb during the GNG task conflicted with previous meta-analyses that included task reactivity (Thayer et al., 2012; Beissner et al., 2013). However, our results mirror previous findings that have also found an inverse relationship between cortical activation and RSA during baseline (Allen et al., 2015; Jennings et al., 2015) and are counter to that found in prior meta-analyses of neuroimaging studies focused on the NVM (Thayer et al., 2012). In the present study, the previous literature was expanded through simultaneous acquisition of RSA and cortical activation data, providing a methodological advance and yielding results consistent with similar to Allen et al. (2015) and Jennings et al. (2015), in which resting cortical and HRV data were collected on separate occasions. Together, these studies suggest that the argument presented in previous meta-analyses of neuroimaging studies focused on the NVM (i.e., that RSA reactivity is positively related to activation of CAN structures during tasks; Thayer et al., 2012) are not representative of the model during rest. It is important to establish the relationship between these variables during a resting state because (1) the NVM is largely centered on resting RSA and its relation to CAN function, and (2) doing so will provide context for how this relationship is altered during reactivity to tasks with varying demands.

Previous studies examining RSA and cortical activation have been conducted at rest or have taken a task-based approach, but few have looked at the metrics simultaneously across both conditions. Of those that have measured RSA and cortical activation simultaneously during tasks (e.g., the n-back task, a speech stressor, a handgrip task, a working memory task), none have used a task involving behavioral inhibition or cognitive flexibility (Gianaros et al., 2004; Thayer et al., 2012; Jennings et al., 2015), although a few have done so using RSA measurements taken outside of the neuroimaging data collection session (Matthews et al., 2004; Neumann et al., 2006; Jennings et al., 2015). The present study used a task that is relevant to the cognitive and behavioral processes implicated in the NVM to determine whether CAN activity (i.e., pre-frontal activation) was still indexed by RSA in this context. The predictive relationship of RSA on mPFC activation during inhibition was not supported in the present study, although a negative relationship was detected at baseline. It is possible that although the relationship is negative at rest, this transitions to a positive relationship during cognitive tasks, as seen in the previous literature, but the behavioral inhibition task used here was not challenging enough to elicit these results. The discrepancy in these findings indicates the need for further investigation of how the NVM applies across varying contexts, as suggested by Jennings et al. (2015) and Smith et al. (2017). It is possible that when CAN structures are recruited for another task, as is the case with the PFC during the GNG, the relationship between these structures and RSA becomes irregular. Understanding this relationship has implications for how behavioral inhibition and flexibility can be assessed through these biological measures, with implications for identifying and tracking inhibition and flexibility deficits.

### Limitations

Although the present study adds to the literature on neuroimaging and the NVM, some limitations should be acknowledged. First, our final sample size (38 individuals) was smaller than the target number recruited for the study (45) due to data loss related to technical problems. Although this sample size is comparable with similar studies (e.g., Chang et al., 2013), we initially aimed for a larger sample to optimize the fNIRS signal-to-noise ratio (Cui et al., 2011). The analyses did not retain statistical significance after Holm–Bonferroni correction for multiple comparisons was applied, although they did at the uncorrected level and were consistent with findings from previous studies. The sole reliance on *p*-values, and consequently, the correcting of *p*-values, is cautioned by the American Statistical Association (Wasserstein and Lazar, 2016). However, the present findings provide preliminary evidence that is incompatible with the null hypothesis that the relation between RMSSD and O_2_Hb is non-existent (e.g., equal to 0), specifically in the mPFC, a finding consistent with previous neuroimaging literature evaluating the NVM. For this reason, continuing to investigate the NVM using fNIRS thorough replications of the present study with increased sample sizes and additional cognitive tasks is warranted.

Additionally, neuroimaging using fNIRS is subject to inherent limitations, which should also be considered in the present study. Although fNIRS has increased spatial resolution to other central nervous system measures, such as electroencephalogram, the spatial resolution is limited compared with fMRI (Irani et al., 2007). fNIRS channels measure cortical activation on the order of centimeters over the cortex, imposing a relative limit on the specificity of activation to certain cortical regions. Additionally, fNIRS can only penetrate approximately 1 cm into the cerebral cortex, limiting which neural structures can be interrogated with this neuroimaging modality. For that reason, subcortical structures in the CAN could not be targeted in the present study. It was with this limitation in mind that hypotheses were specifically written regarding the mPFC, allowing for a component of the CAN to be assessed while taking advantage of the flexibilities afforded by an fNIRS study (e.g., measuring brain activation and RSA while sitting upright, as is more common in resting-state psychophysiological assessment).

Further, the present analyses were largely based on previous fMRI studies that examined the NVM through traditional fMRI analysis procedures (Chang et al., 2013). Although these methods are standard in the field, recent work suggests that there are more advanced statistical approaches that can be applied to imaging data. A majority of the fMRI literature utilizes a summary statistic approach, wherein subject-level regressions are conducted at the voxel level, and the summary statistics (i.e., β estimates) resulting from these analyses are used to conduct group-level analyses (Monti, 2011). The problem with this approach is that it does not account for individual variances from the subject-level equations when conducting group-level analyses. More recently, fMRI researchers have been advocating for the use of more comprehensive statistical approaches, such as the “sufficient-summary-statistic approach” (Dowding and Haufe, 2018) or the use of hierarchical/multilevel mixed linear models (Chen et al., 2013). In the present study, the analyses were intentionally modeled after previous fMRI research because multiple hypotheses and corresponding research design elements were already being introduced (e.g., the use of fNIRS, adding multiple conditions to examine research questions examining the NVM). However, more robust modeling approaches should be considered when examining these research questions moving forward.

### Future Directions

In addition to the advanced statistical modeling approaches mentioned earlier, several other methodologies should be used, and other research questions pursued in relation to our findings. First, PFC activation in relation to RSA should be assessed across additional cognitive tasks related to behavioral inhibition and cognitive flexibility. For example, in addition to replicating the present study with more complex versions of the GNG task, various versions of the Stroop Color–Word task could be used to examine inhibition of cognitive interference (Scarpina and Tagini, 2017) and other task-switching or set-shifting paradigms (Dajani and Uddin, 2015). Although a relationship between RSA and pre-frontal activation during the GNG task was not found in the present study, it is possible that this could be due to task demands. A simple version of the GNG was used, which may not have required the mobilization of many cognitive resources. With a task requiring a higher cognitive load, there may be different results. For example, Gianaros et al. (2004) noted the use of increasingly complex working memory tasks in their design, resulting in differences in the task-related findings. The present study used a simple GNG design, as GNG tasks with increased demands have been critiqued for drawing on cognitive functions beyond behavior inhibition alone, which are not as relevant to the NVM. However, assessing the relationship between PFC activation and RSA across various aspects of cognitive flexibility and at varying levels of a cognitive load will help validate whether the biological principles behind the NVM generalize across contexts. Such investigations could reveal whether these metrics differentially relate to various components of cognitive flexibility and have important implications for how these biological metrics could be utilized for individuals with deficits in cognitive flexibility.

## Conclusion

The present study examined pre-frontal activation during rest and a behavioral inhibition task through fNIRS, used simultaneous assessment of neural and parasympathetic output to improve upon prior evaluations of the NVM, and attempted to relate task performance, neuroimaging, and RSA measures during behavioral inhibition to deficits in other areas related to behavioral and cognitive flexibility. The present findings not only inform theoretical aspects of the NVM but also speak to broader applications of the model to other domains of functioning. From a methodological perspective, the present study provides valuable information regarding the use of fNIRS in conjunction with RSA to evaluate the NVM. The findings using fNIRS are consistent with previous studies that have used fMRI to investigate research questions surrounding the model and behavior inhibition. Furthermore, the present study indicates that fNIRS is a viable alternative to fMRI, which is comparatively more cumbersome and expensive, in assessing these research questions. From a theoretical perspective, the present study provides information about the NVM beyond the previous literature through assessment at baseline and during behavior inhibition.

Specifically, the findings indicate that the relationship between RSA and pre-frontal activation proposed in the model is likely to vary depending on environmental demands. These findings indicate the importance of assessing the NVM across multiple contexts moving forward and, consequently, provide insight for how these measures may be applied in addressing behavioral flexibility deficits. Incorporating these considerations into future studies will be imperative in assessing the practicability of using the biological markers proposed in the NVM as potential therapeutic targets or evaluation tools for these deficits.

## Data Availability Statement

The raw data supporting the conclusions of this article will be made available by the authors, without undue reservation.

## Ethics Statement

The studies involving human participants were reviewed and approved by the National Institute of Child Health and Human Development Institutional Review Board. The patients/participants provided their written informed consent to participate in this study.

## Author Contributions

EC, BF, and AG were responsible for developing the theoretical background, analytical plan, and drafting the manuscript. EC was responsible for data collection and data analysis. All authors listed have made a substantial, direct and intellectual contribution to the work, and approved it for publication.

## Conflict of Interest

The authors declare that the research was conducted in the absence of any commercial or financial relationships that could be construed as a potential conflict of interest.

## Footnotes

1 A third enteric branch, which innervates the gastrointestinal organs, is sometimes also distinguished (see Janig and Habler, 2000).

## References

[B1] Aasted C. M. Yucel M. A. Cooper R. J. Dubb J. Tsuzuki D. Becerra L. (2015). Anatomical guidance for functional near-infrared spectroscopy: atlas viewer tutorial. *Neurophotonics* 2:020801 10.1117/1.NPh.2.2.020801 PMC4478785 26157991

[B2] Allen B. Jennings J. R. Gianaros P. J. Thayer J. F. Manuck S. B. (2015). Resting high-frequency heart rate variability is related to resting brain perfusion. *Psychophysiology* 52 277–287. 10.1111/psyp.12321 25174686 PMC4387872

[B3] Allen J. J. Chambers A. S. Towers D. N. (2007). The many metrics of cardiac chronotropy: a pragmatic primer and a brief comparison of metrics. *Biol. Psychol.* 74 243–262. 10.1016/j.biopsycho.2006.08.005 17070982

[B4] Anderson A. A. Smith E. Chernomordik V. Ardeshirpour Y. Chowdhry F. Thurm A. (2014). Prefrontal cortex hemodynamics and age: a pilot study using functional near infrared spectroscopy in children. *Front. Neurosci.* 8:393. 10.3389/fnins.2014.00393 25565935 PMC4266015

[B5] Ayaz H. Shewokis P. A. Curtin A. Izzetoglu M. Izzetoglu K. Onaral B. (2011). Using MazeSuite and functional near infrared spectroscopy to study learning in spatial navigation. *J. Vis. Exp.* 56:3443. 10.3791/3443 22005455 PMC3227178

[B6] Beissner F. Meissner K. Bar K. J. Napadow V. (2013). The autonomic brain: an activation likelihood estimation meta-analysis for central processing of autonomic function. *J. Neurosci.* 33 10503–10511. 10.1523/JNEUROSCI.1103-13.2013 23785162 PMC3685840

[B7] Benarroch E. E. (1993). The central autonomic network: functional organization, dysfunction, and perspective. *Mayo. Clin. Proc.* 68 988–1001. 10.1016/s0025-6196(12)62272-1 8412366

[B8] Berntson G. G. Bigger J. T. Jr. Eckberg D. L. Grossman P. Kaufmann P. G. Malik M. (1997). Heart rate variability: origins, methods, and interpretive caveats. *Psychophysiology* 34 623–648. 10.1111/j.1469-8986.1997.tb02140.x 9401419

[B9] Berntson G. G. Cacioppo J. T. Quigley K. S. (1993). Respiratory sinus arrhythmia: autonomic origins, physiological mechanisms, and psychophysiological implications. *Psychophysiology* 30 183–196. 10.1111/j.1469-8986.1993.tb01731.x 8434081

[B10] Brigadoi S. Ceccherini L. Cutini S. Scarpa F. Scatturin P. Selb J. (2014). Motion artifacts in functional near-infrared spectroscopy: a comparison of motion correction techniques applied to real cognitive data. *Neuroimage* 85(Pt 1), 181–191. 10.1016/j.neuroimage.2013.04.082 23639260 PMC3762942

[B11] Buckner R. L. Andrews-Hanna J. R. Schacter D. L. (2008). The brain’s default network: anatomy, function, and relevance to disease. *Ann. N Y Acad. Sci.* 1124 1–38. 10.1196/annals.1440.011 18400922

[B12] Chang C. Metzger C. D. Glover G. H. Duyn J. H. Heinze H. J. Walter M. (2013). Association between heart rate variability and fluctuations in resting-state functional connectivity. *Neuroimage* 68 93–104. 10.1016/j.neuroimage.2012.11.038 23246859 PMC3746190

[B13] Chen G. Saad Z. S. Britton J. C. Pine D. S. Cox R. W. (2013). Linear mixed-effects modeling approach to FMRI group analysis. *Neuroimage* 73 176–190. 10.1016/j.neuroimage.2013.01.047 23376789 PMC3638840

[B14] Cui X. Bray S. Bryant D. M. Glover G. H. Reiss A. L. (2011). A quantitative comparison of NIRS and fMRI across multiple cognitive tasks. *Neuroimage* 54 2808–2821. 10.1016/j.neuroimage.2010.10.069 21047559 PMC3021967

[B15] Dajani D. R. Uddin L. Q. (2015). Demystifying cognitive flexibility: Implications for clinical and developmental neuroscience. *Trends Neurosci.* 38 571–578. 10.1016/j.tins.2015.07.003 26343956 PMC5414037

[B16] Dillo W. Goke A. Prox-Vagedes V. Szycik G. R. Roy M. Donnerstag F. (2010). Neuronal correlates of ADHD in adults with evidence for compensation strategies–a functional MRI study with a Go/No-Go paradigm. *Ger. Med. Sci.* 8:Doc09. 10.3205/000098 20421953 PMC2858877

[B17] Donders F. C. (1969). On the speed of mental processes. *Acta. Psychol.* 30 412–431. 10.1016/0001-6918(69)90065-1 5811531

[B18] Dowding I. Haufe S. (2018). Powerful Statistical Inference for Nested Data Using Sufficient Summary Statistics. *Front. Hum. Neurosci.* 12:103. 10.3389/fnhum.2018.00103 29615885 PMC5867457

[B19] Eatough E. M. Shirtcliff E. A. Hanson J. L. Pollak S. D. (2009). Hormonal reactivity to MRI scanning in adolescents. *Psychoneuroendocrinology* 34 1242–1246. 10.1016/j.psyneuen.2009.03.006 19346079 PMC2747582

[B20] Ferrari M. Quaresima V. (2012). A brief review on the history of human functional near-infrared spectroscopy (fNIRS) development and fields of application. *Neuroimage* 63 921–935. 10.1016/j.neuroimage.2012.03.049 22510258

[B21] Fox P. T. Raichle M. E. (1986). Focal physiological uncoupling of cerebral blood flow and oxidative metabolism during somatosensory stimulation in human subjects. *Proc. Natl. Acad. Sci.* 83 1140–1144. 10.1073/pnas.83.4.1140 3485282 PMC323027

[B22] Friedman B. H. (2007). An autonomic flexibility-neurovisceral integration model of anxiety and cardiac vagal tone. *Biol. Psychol.* 74 185–199. 10.1016/j.biopsycho.2005.08.009 17069959

[B23] Gianaros P. J. Van Der Veen F. M. Jennings J. R. (2004). Regional cerebral blood flow correlates with heart period and high-frequency heart period variability during working-memory tasks: Implications for the cortical and subcortical regulation of cardiac autonomic activity. *Psychophysiology* 41 521–530. 10.1111/1469-8986.2004.00179.x 15189475 PMC4301264

[B24] Herrmann M. J. Plichta M. M. Ehlis A. C. Fallgatter A. J. (2005). Optical topography during a Go-NoGo task assessed with multi-channel near-infrared spectroscopy. *Behav. Brain Res.* 160 135–140. 10.1016/j.bbr.2004.11.032 15836908

[B25] Irani F. Platek S. M. Bunce S. Ruocco A. C. Chute D. (2007). Functional near infrared spectroscopy (fNIRS): an emerging neuroimaging technology with important applications for the study of brain disorders. *Clin. Neuropsychol.* 21 9–37. 10.1080/13854040600910018 17366276

[B26] Janig W. Habler H. J. (2000). Specificity in the organization of the autonomic nervous system: a basis for precise neural regulation of homeostatic and protective body functions. *Prog. Brain Res.* 122 351–367. 10.1016/s0079-6123(08)62150-0 10737070

[B27] Jennings J. R. Allen B. Gianaros P. J. Thayer J. F. Manuck S. B. (2015). Focusing neurovisceral integration: cognition, heart rate variability, and cerebral blood flow. *Psychophysiology* 52 214–224. 10.1111/psyp.12319 25160649 PMC4387874

[B28] Jennings J. R. Kamarck T. Stewart C. Eddy M. Johnson P. (1992). Alternate cardiovascular baseline assessment techniques: vanilla or resting baseline. *Psychophysiology* 29 742–750. 10.1111/j.1469-8986.1992.tb02052.x 1461961

[B29] Laborde S. Mosley E. Thayer J. F. (2017). Heart rate variability and cardiac vagal tone in psychophysiological research–recommendations for experiment planning, data analysis, and data reporting. *Front. Psychol.* 8:213. 10.3389/fpsyg.2017.00213 28265249 PMC5316555

[B30] Lee H. J. Yost B. P. Telch M. J. (2009). Differential performance on the go/no-go task as a function of the autogenous-reactive taxonomy of obsessions: findings from a non-treatment seeking sample. *Behav. Res. Ther.* 47 294–300. 10.1016/j.brat.2009.01.002 19217612

[B31] Lloyd-Fox S. Blasi A. Elwell C. E. (2010). Illuminating the developing brain: the past, present and future of functional near infrared spectroscopy. *Neurosci. Biobehav. Rev.* 34 269–284. 10.1016/j.neubiorev.2009.07.008 19632270

[B32] Lueken U. Muehlhan M. Evens R. Wittchen H. U. Kirschbaum C. (2012). Within and between session changes in subjective and neuroendocrine stress parameters during magnetic resonance imaging: A controlled scanner training study. *Psychoneuroendocrinology* 37 1299–1308. 10.1016/j.psyneuen.2012.01.003 22309826

[B33] Matthews S. C. Paulus M. P. Simmons A. N. Nelesen R. A. Dimsdale J. E. (2004). Functional subdivisions within anterior cingulate cortex and their relationship to autonomic nervous system function. *Neuroimage* 22 1151–1156. 10.1016/j.neuroimage.2004.03.005 15219587

[B34] Monti M. M. (2011). Statistical Analysis of fMRI Time-Series: A Critical Review of the GLM Approach. *Front. Hum. Neurosci.* 5:28. 10.3389/fnhum.2011.00028 21442013 PMC3062970

[B35] Mostofsky S. H. Schafer J. G. Abrams M. T. Goldberg M. C. Flower A. A. Boyce A. (2003). fMRI evidence that the neural basis of response inhibition is task-dependent. *Brain Res. Cogn. Brain. Res.* 17 419–430. 10.1016/s0926-6410(03)00144-7 12880912

[B36] Munoz M. L. van Roon A. Riese H. Thio C. Oostenbroek E. Westrik I. (2015). Validity of (Ultra-)Short Recordings for Heart Rate Variability Measurements. *PLoS One* 10:e0138921. 10.1371/journal.pone.0138921 26414314 PMC4586373

[B37] Neumann S. A. Brown S. M. Ferrell R. E. Flory J. D. Manuck S. B. Hariri A. R. (2006). Human choline transporter gene variation is associated with corticolimbic reactivity and autonomic-cholinergic function. *Biol. Psychiatry.* 60 1155–1162. 10.1016/j.biopsych.2006.03.059 16876130

[B38] Oldfield R. C. (1971). The assessment and analysis of handedness: the Edinburgh inventory. *Neuropsychologia* 9 97–113. 10.1016/0028-3932(71)90067-4 5146491

[B39] R Core Team (2017). *R: A Language and Environment for Statistical Computing.* Vienna: R Foundation for Statistical Computing.

[B40] Raichle M. E. (2015). The brain’s default mode network. *Annu. Rev. Neurosci.* 38 433–447. 10.1146/annurev-neuro-071013-014030 25938726

[B41] Rodrigo A. H. Domenico S. I. Ayaz H. Gulrajani S. Lam J. Ruocco A. C. (2014). Differentiating functions of the lateral and medial prefrontal cortex in motor response inhibition. *Neuroimage* 85(Pt 1), 423–431. 10.1016/j.neuroimage.2013.01.059 23384524

[B42] Saul J. P. (1990). Beat-to-Beat Variations of Heart-Rate Reflect Modulation of Cardiac Autonomic Outflow. *News Physiol. Sci.* 5 32–37. 10.1152/physiologyonline.1990.5.1.32

[B43] Scarpina F. Tagini S. (2017). The Stroop Color and Word Test. *Front. Psychol.* 8:557. 10.3389/fpsyg.2017.00557 28446889 PMC5388755

[B44] Schmidt B. (2006). Proof of principle studies. *Epilep. Res.* 68 48–52. 10.1016/j.eplepsyres.2005.09.019 16377153

[B45] Schneider W. Eschman A. Zuccolotto A. (2002). *E-Prime User’s Guide.* Pittsburgh: Psychology Software Tools Inc.

[B46] Schulz K. P. Fan J. Magidina O. Marks D. J. Hahn B. Halperin J. M. (2007). Does the emotional go/no-go task really measure behavioral inhibition? Convergence with measures on a non-emotional analog. *Arch. Clin. Neuropsychol.* 22 151–160. 10.1016/j.acn.2006.12.001 17207962 PMC2562664

[B47] Smith R. Thayer J. F. Khalsa S. S. Lane R. D. (2017). The hierarchical basis of neurovisceral integration. *Neurosci. Biobehav. Rev.* 75 274–296. 10.1016/j.neubiorev.2017.02.003 28188890

[B48] Swick D. Ashley V. Turken U. (2011). Are the neural correlates of stopping and not going identical? Quantitative meta-analysis of two response inhibition tasks. *Neuroimage* 56 1655–1665. 10.1016/j.neuroimage.2011.02.070 21376819

[B49] Takahashi N. Kuriyama A. Kanazawa H. Takahashi Y. Nakayama T. (2017). Validity of spectral analysis based on heart rate variability from 1-minute or less ECG recordings. *Pacing Clin. Electrophysiol.* 40 1004–1009. 10.1111/pace.13138 28594089

[B50] Tarvainen M. P. Niskanen J. P. Lipponen J. A. Ranta-Aho P. O. Karjalainen P. A. (2014). Kubios HRV–heart rate variability analysis software. *Comput. Methods Programs Biomed.* 113 210–220. 10.1016/j.cmpb.2013.07.024 24054542

[B51] Task Force (1996). Heart rate variability. Standards of measurement, physiological interpretation, and clinical use. Task Force of the European Society of Cardiology and the North American Society of Pacing and Electrophysiology. *Eur. Heart J.* 17 354–381. 8737210

[B52] Thayer J. F. Ahs F. Fredrikson M. Sollers J. J. III Wager T. D. (2012). A meta-analysis of heart rate variability and neuroimaging studies: implications for heart rate variability as a marker of stress and health. *Neurosci. Biobehav. Rev.* 36 747–756. 10.1016/j.neubiorev.2011.11.009 22178086

[B53] Thayer J. F. Hansen A. L. Saus-Rose E. Johnsen B. H. (2009). Heart rate variability, prefrontal neural function, and cognitive performance: the neurovisceral integration perspective on self-regulation, adaptation, and health. *Ann. Behav. Med.* 37 141–153. 10.1007/s12160-009-9101-z 19424767

[B54] Thayer J. F. Friedman B. H. (2002). Stop that! inhibition, sensitization, and their neurovisceral concomitants. *Scand. J. Psychol.* 43 123–130. 10.1111/1467-9450.00277 12004949

[B55] Thayer J. F. Lane R. D. (2000). A model of neurovisceral integration in emotion regulation and dysregulation. *J. Affect Disord.* 61 201–216. 10.1016/s0165-0327(00)00338-4 11163422

[B56] Thayer J. F. Lane R. D. (2009). Claude Bernard and the heart-brain connection: further elaboration of a model of neurovisceral integration. *Neurosci. Biobehav. Rev.* 33 81–88. 10.1016/j.neubiorev.2008.08.004 18771686

[B57] Turner H. M. I. Bernard R. M. (2006). Calculating and synthesizing effect sizes. *Contempor. Issues Commun. Sci. Disord.* 33 42–55. 10.1044/cicsd_33_s_42

[B58] Uzefovsky F. Allison C. Smith P. Baron-Cohen S. (2016). Brief Report: The Go/No-Go Task Online: Inhibitory Control Deficits in Autism in a Large Sample. *J. Autism. Dev. Disord.* 46 2774–2779. 10.1007/s10803-016-2788-3 27103120 PMC4938852

[B59] Wasserstein R. L. Lazar N. A. (2016). The ASA’s Statement on p-Values: Context. *Process Purp. Am. Statist.* 70 129–131. 10.1080/00031305.2016.1154108

[B60] Watanabe J. Sugiura M. Sato K. Sato Y. Maeda Y. Matsue Y. (2002). The human prefrontal and parietal association cortices are involved in NO-GO performances: an event-related fMRI study. *Neuroimage* 17 1207–1216. 10.1006/nimg.2002.1198 12414261

[B61] Yerys B. E. Jankowski K. F. Shook D. Rosenberger L. R. Barnes K. A. Berl M. M. (2009). The fMRI success rate of children and adolescents: typical development, epilepsy, attention deficit/hyperactivity disorder, and autism spectrum disorders. *Hum. Brain Mapp.* 30 3426–3435. 10.1002/hbm.20767 19384887 PMC2748172

